# Sphingosine kills bacteria by binding to cardiolipin

**DOI:** 10.1074/jbc.RA119.012325

**Published:** 2020-04-23

**Authors:** Rabea Verhaegh, Katrin Anne Becker, Michael J. Edwards, Erich Gulbins

**Affiliations:** 1 Department of Molecular Biology, University Clinic, University of Duisburg-Essen, Essen, Germany; 2 Department of Surgery, College of Medicine, University of Cincinnati, Cincinnati, Ohio, USA

**Keywords:** cardiolipin, Pseudomonas aeruginosa (P. aeruginosa), Staphylococcus aureus (S. aureus), sphingolipid, innate immunity, bactericidal activity, lung pathogen, membrane permeabilization, sphingoid base, sphingosine

## Abstract

Sphingosine is a long-chain sphingoid base that has been shown to have bactericidal activity against many pathogens, including *Pseudomonas aeruginosa*, *Staphylococcus aureus,* and *Escherichia coli*. We have previously demonstrated that sphingosine is present in nasal, tracheal, and bronchial epithelial cells and constitutes a central element of the defense of the airways against bacterial pathogens. Here, using assorted lipid-binding and cell biology assays, we demonstrate that exposing *P. aeruginosa* and *S. aureus* cells to sphingosine results in a very rapid, *i.e.* within minutes, permeabilization of the bacterial plasma membrane, resulting in leakiness of the bacterial cells, loss of ATP, and loss of bacterial metabolic activity. These alterations rapidly induced bacterial death. Mechanistically, we demonstrate that the presence of the protonated NH_2_ group in sphingosine, which is an amino-alcohol, is required for sphingosine's bactericidal activity. We also show that the protonated NH_2_ group of sphingosine binds to the highly negatively–charged lipid cardiolipin in bacterial plasma membranes. Of note, this binding was required for bacterial killing by sphingosine, as revealed by genetic experiments indicating that *E. coli* or *P. aeruginosa* strains that lack cardiolipin synthase are resistant to sphingosine, both *in vitro* and *in vivo*. We propose that binding of sphingosine to cardiolipin clusters cardiolipin molecules in the plasma membrane of bacteria. This clustering results in the formation of gel-like or even crystal-like structures in the bacterial plasma membrane and thereby promotes rapid permeabilization of the plasma membrane and bacterial cell death.

## Introduction

*Pseudomonas aeruginosa* is a ubiquitous and opportunistic pathogen that causes severe respiratory tract and systemic infections, especially among patients with cystic fibrosis (CF) and chronic obstructive pulmonary disorders (COPD), previous viral infections, burn wounds, trauma or sepsis, and those patients requiring mechanical ventilation (1–6). Most important are the *P. aeruginosa* and *Staphylococcus aureus* infections among patients with CF. CF, which is caused by mutations of the cystic fibrosis transmembrane conductance regulator gene (human, *CFTR*; murine, *Cftr*) (7, 8), is the most common recessively-inherited disorder in North America and Europe, with more than 80,000 CF patients in the EU and the USA alone (9). The most frequent cause of morbidity and mortality among CF patients is chronic pulmonary infection with bacterial pathogens, in particular *P. aeruginosa* and *S. aureus* (*e.g.* 10, 11). Approximately 80% of all CF patients are colonized with *P. aeruginosa* by the age of 25. At present, no treatment to eradicate these bacterial infections is available.

Many *P. aeruginosa* and *S. aureus* strains are highly resistant to existing antibiotics, and attempts to eradicate pulmonary *P. aeruginosa* or *S. aureus* among CF or COPD patients usually fail. Thus, it is important to develop novel strategies for treating pulmonary infections with *P. aeruginosa* and *S. aureus*.

Sphingolipids are a class of lipids that share an amino alcohol (sphingoid base) backbone. We have previously demonstrated that sphingosine efficiently kills many bacterial species *in vitro* and *in vivo*, including *P. aeruginosa*, *S. aureus* (even methicillin-resistant *S. aureus*), *Acinetobacter baumannii*, *Haemophilus influenzae*, *Burkholderia cepacia,* and *Moraxella catarrhalis* (12, 13). Other groups have shown that sphingosine also kills *Escherichia coli* and *Porphyromonas gingivalis* (14–17).

Our previous studies also demonstrated that sphingosine is an abundant constituent of the luminal surface of human nasal, tracheal, and bronchial epithelial cells obtained from healthy subjects and from epithelial cells of the trachea and conducting bronchi of WT mice, whereas it is almost undetectable on the surface of nasal, tracheal, and bronchial epithelial cells from CF patients and on tracheal and bronchial cells from CF mice (12, 13, 18). Treating CF mice with inhaled sphingosine eliminated existing acute and chronic pulmonary *P. aeruginosa* infections and prevented new *P. aeruginosa* or *S. aureus* infections in these mice (12, 13, 18), a finding demonstrating that sphingosine plays a key role in the innate and immediate defense of the upper respiratory tract.

Likewise, the inhalation of recombinant human acid ceramidase by CF mice restored epithelial airway sphingosine levels and reversed acute and chronic infection with *P. aeruginosa* (12, 18).

These studies demonstrate a central role of sphingosine in the defense against bacterial pathogens in pulmonary infections.

EM studies by Fischer *et al.* (15) indicated that killing of pathogens by sphingosine does not result in simple lysis of pathogens such as Gram-positive *S. aureus* and Gram-negative *E. coli*.

These studies clearly indicate that sphingosine is bactericidal for a variety of pathogens, but the molecular mechanisms of sphingosine-mediated killing of bacteria remain to be defined.

Here, we demonstrate that sphingosine binds to cardiolipin in the bacterial plasma membrane resulting in rapid permeabilization of the bacterial membrane and death. Bacterial mutants that lack cardiolipin are resistant to sphingosine-mediated toxicity *in vitro* and *in vivo*. Thus, the interaction of sphingosine with cardiolipin is required for its bactericidal effects. Sphingosine most likely acts by inducing very rigid cardiolipin–membrane domains, a process that results in leakiness of the membrane and bacterial death. This notion is consistent with biophysical experiments measuring membrane fluidity in liposomes after treatment with sphingosine.

## Results

**Figure 1.**
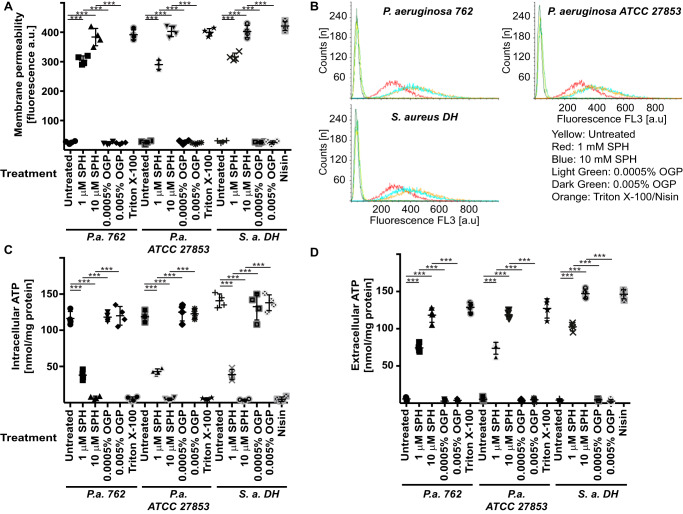
**Sphingosine induces a rapid increase in the permeability of *P. aeruginosa*.** *A* and *B, P. aeruginosa* (*P.a.*) strains 762 and ATCC 27853 or *S. aureus* (*S.a.*) strain DH was incubated with 100 nm TO-PRO-3 iodide. Bacteria were then treated for 5 min with 1 or 10 μm sphingosine (*SPH*) or the corresponding concentration of octylglucopyranoside (*OGP*), the solvent of sphingosine. The bacteria were analyzed by flow cytometry. *A* shows the quantitative analysis of the means of the fluorescence intensity obtained in the flow cytometry studies (given in arbitrary units; *a.u.*), and *B* shows representative flow cytometry stainings from four independent experiments. Either Triton X-100 or nisin was added as a positive control for membrane permeabilization. *C* and *D*, intracellular ATP in *P. aeruginosa* strains 762 and ATCC 27853 or *S. aureus* strain DH (*C*) and the release of ATP (*D*) from the bacteria were measured with the BacTiter-Glo reagent. SPH results in a very rapid release of ATP from *P. aeruginosa* or *S. aureus* into the supernatant. OGP, the solvent of sphingosine, added at the same concentrations as in the samples with sphingosine, exerted no effect. Either Triton or nisin was added as a positive control for membrane permeabilization and ATP release into the medium. Displayed are the means ± S.D. of four independent experiments each in *A*, *C*, and *D*. ***, *p* < 0.001, ANOVA.

To define molecular mechanisms that mediate the bactericidal effects of sphingosine, we incubated two *P. aeruginosa* strains, *i.e.* the clinical isolate 762 and the laboratory strain American Type Culture Collection (ATCC) 27853, and a clinical isolate of *S. aureus*, named DH, with 1 or 10 μm sphingosine or the corresponding concentrations of octylglucopyranoside, the detergent used to solubilize sphingosine. The bacteria were incubated with sphingosine for 5 min. We then determined the membrane permeability of the bacteria by staining with TO-PRO^TM^-3 iodide. Viable cells are impermeable to TO-PRO-3, which binds to chromosomes after an increase in membrane permeability. This can be visualized by fluorescence microscopy or flow cytometry. The data show that incubation of *P. aeruginosa* or *S. aureus* with sphingosine results in a rapid increase in bacterial permeability (Fig. 1, *A* and *B*). An additional time-course study showed that sphingosine mediates almost complete cell permeability already within 5 min, resulting in increased staining of the bacteria with TO-PRO-3 (data not shown).

To confirm the rapid effect of sphingosine on the viability of bacteria, we determined the release of ATP into the medium upon incubation of *P. aeruginosa* and *S. aureus* with sphingosine. The results showed a rapid release of ATP into the medium (Fig. 1, *C* and *D*).

**Figure 2.**
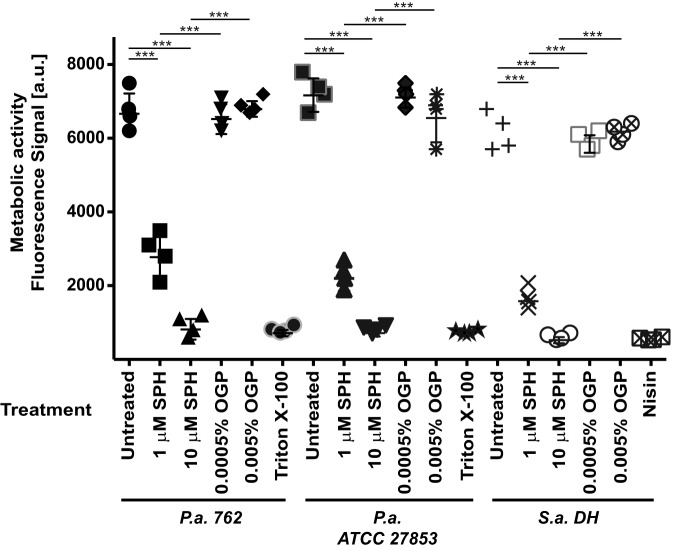
**Sphingosine abrogates the metabolic activity of *P. aeruginosa* and *S. aureus*.** *P. aeruginosa* (*P.a.*) strains 762 and American Type Culture Collection (ATCC) 27853 or *S. aureus* (*S.a.*) strain DH was treated with 1 or 10 μm SPH or OGP, the solvent of sphingosine, at the corresponding concentrations. Samples were then incubated with the oxidized, nonfluorescent form of C_12_-resazurin, and the metabolic activity was determined with flow cytometry by measuring the generation of fluorescent C_12_-resorufin. The studies showed that sphingosine results in a very rapid decrease in bacterial metabolic activity. Either Triton or nisin was added as a positive control for membrane permeabilization and disruption of bacterial metabolism. Fluorescence intensity is given in arbitrary units (*a.u.*). Displayed are the means ± S.D. of four independent experiments each. ***, *p* < 0.001, ANOVA.

In accordance with the massive increase of membrane permeability upon incubation of the bacteria with sphingosine, we also observed a dramatic decrease in metabolic activity in *P. aeruginosa* or *S. aureus* upon incubation with sphingosine (Fig. 2).

**Figure 3.**
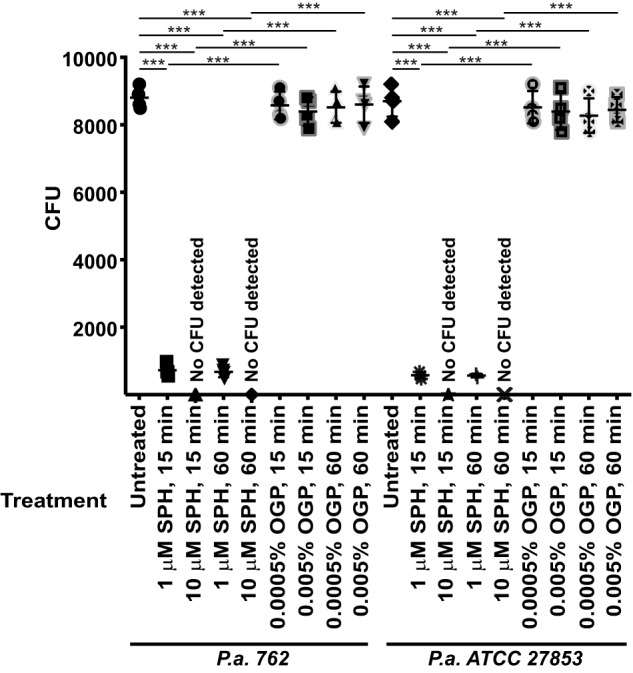
**Sphingosine mediates killing of bacteria within minutes.** Each of the 10,000 cfu of *P. aeruginosa* (*P.a.*) strains 762 and ATCC 27853 was incubated in PBS (pH 7.0) with 1 or 10 μm SPH or the corresponding concentrations of the solvent OGP for 15 or 60 min. Samples were washed, and aliquots were plated on trypticase soy broth plates and were counted after growth overnight. Shown are the means ± S.D. of four independent experiments each. ***, *p* < 0.001, ANOVA.

Next, we aimed to further define the time course of sphingosine-mediated killing of *P. aeruginosa*. To this end, we incubated the *P. aeruginosa* strains 762 or ATCC 27853 with 1 or 10 μm sphingosine for 15 or 60 min, washed, and re-cultured the bacteria. The results of these studies demonstrate that sphingosine killed the bacteria within 15 min (Fig. 3), a finding consistent with the very rapid action of sphingosine on membrane integrity/permeability.

Sphingosine contains an NH_2_ group and an OH group. At neutral or slightly acidic pH, as found in airways and on many epithelial cell surfaces (19), the NH_2_ group will be protonated and thus positively charged. Therefore, we first tested whether the NH_2_ group is important, and second whether protonation of this group is required for the bactericidal effects of sphingosine.

**Figure 4.**
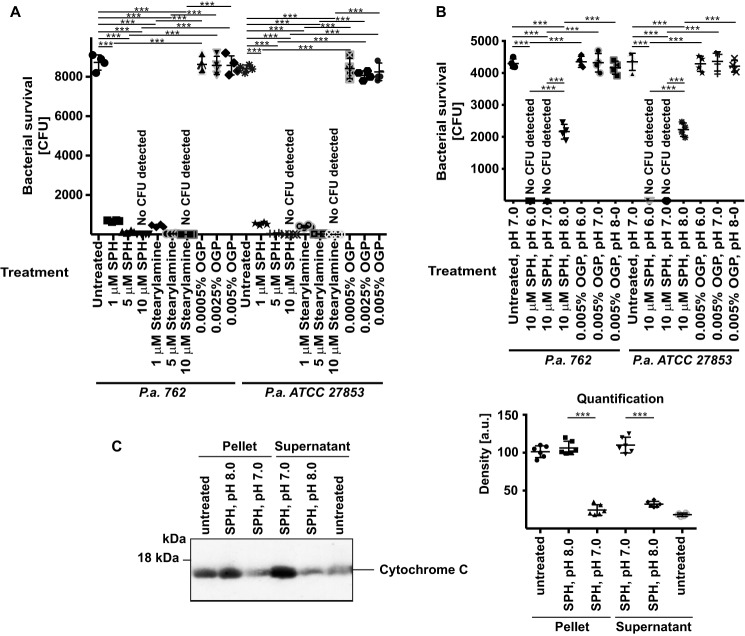
**NH_2_ group and protonation of this group are required for the bactericidal effects of sphingosine on *P. aeruginosa*.** *A*, each of the 10,000 cfu of *P. aeruginosa* (*P.a.*) strains 762 and ATCC 27853 was incubated with 1, 5, or 10 μm stearylamine or SPH or 0.0005, 0.0025, or 0.005% of the solvent OGP in PBS adjusted to pH 7.0. After 60 min, the bacteria were washed; aliquots were plated on trypticase soy broth plates, and cfu were counted after growth overnight. Shown are the means ± S.D. of four independent experiments each. ***, *p* < 0.001, ANOVA. *B*, each of the 10,000 cfu of *P. aeruginosa* (*P.a.*) strains 762 and ATCC 27853 was incubated with 10 μm SPH in PBS adjusted to a pH of 6.0, 7.0, or 8.0 for 60 min and then washed. Controls were incubated with the corresponding concentrations of the solvent OGP. Aliquots were then plated on trypticase soy broth plates, and cfu were counted after growth overnight. Shown are the means ± S.D. of four independent experiments each. ***, *p* < 0.001, ANOVA. *C*, isolated mitochondria were incubated with 0.5 μm sphingosine at pH 7.0 or 8.0 for 30 min on ice or left untreated. The results indicate a release of cytochrome *c* from isolated mitochondria at pH 7.0, whereas sphingosine is inactive at pH 8.0. Shown is a representative Western blotting demonstrating the release of cytochrome *c* into the supernatant and the amount of cytochrome *c* in the corresponding mitochondrial pellets from four independent experiments and the quantitative analysis using Photoshop® for measuring the density of the Western blotting signals. Given are the means ± S.D., *n* = 4; ***, *p* < 0.001; *t* test.

First, to test whether the amine group mediates the bactericidal effects of sphingosine, we incubated *P. aeruginosa* with 1 or 10 μm stearylamine, which is structurally very similar to sphingosine but lacks the OH group. The results showed that stearylamine kills *P. aeruginosa* as efficiently as sphingosine (Fig. 4*A*).

Second, to test whether the pH determines the effects of sphingosine on *P. aeruginosa*, we measured whether an increase of the pH from 6.0 to 8.0 alters the bactericidal effects of sphingosine. The results show that the effects of sphingosine on *P. aeruginosa* are greatly reduced at pH 8.0 (Fig. 4*B*). This finding indicates that protonation of the NH_2_ group is necessary for the antibacterial effects of sphingosine.

To further confirm the notion of an interaction of sphingosine with cardiolipin, we tested whether sphingosine induces a release of cytochrome *c* from isolated mitochondria. The data (Fig. 4*C*) show that sphingosine induces a release of cytochrome *c* from mitochondria, which is prevented by alkalization of the samples.

Given (i) the structure of sphingosine, which is an amino alcohol, (ii) the obvious significance of the NH_2_ group for sphingosine-mediated killing of bacteria, and (iii) the observation that bacteria are much more sensitive to sphingosine than mammalian cells (12, 13, 18), we hypothesized that the NH_2_ group in sphingosine becomes protonated at a neutral or slightly acidic pH and binds to negatively-charged molecules in the plasma membrane of bacteria. One candidate meeting the characteristics of such a molecule is cardiolipin, a negatively-charged lipid that is very important for respiration. Cardiolipin is present in the plasma membrane of bacteria, but it is absent from the plasma membrane of mammalian cells, in which it is present exclusively in mitochondrial membranes (consistent with the endosymbiont hypothesis).

**Figure 5.**
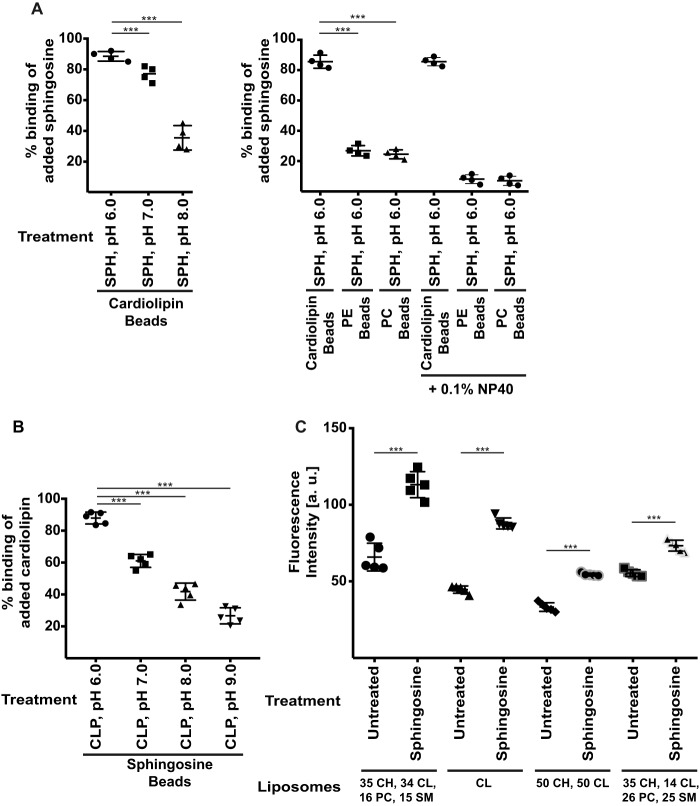
**Sphingosine binds to cardiolipin.** *A*, agarose-bound cardiolipin or control phosphatidylethanolamine (*PE*) and phosphatidylcholine (*PC*) beads were co-incubated with 1 nmol of SPH at the indicated pH, extensively washed, and extracted, and the amount of bound sphingosine was determined by a sphingosine kinase assay. The solvent octylglucopyranoside (used as a control; not shown) showed no background binding to cardiolipin in the kinase assays. Addition of 0.1% NP-40 removed the residual binding of sphingosine to phosphatidylethanolamine and phosphatidylcholine beads. Shown are the means ± S.D. from four independent experiments and each of the percentage of the added 1 nmol of SPH that was bound to cardiolipin; ***, *p* < 0.001; ANOVA. *B*, agarose-bound sphingosine or control beads were co-incubated with 2 nmol of cardiolipin (*CLP*) and extensively washed, and the amount of bound cardiolipin was measured with a fluorescence assay. Sphingosine beads showed no background in the assay. Shown are the means ± S.D. from five experiments each of the percentage of the added 2 nmol of CLP that was bound to cardiolipin. *C*, membrane fluidity of different liposomes prior to and after addition of sphingosine was measured by a fluorescence assay using the fluorescent probe DPH. The lipid composition is indicated in the figure in mol %. Shown are means ± S.D. from five independent experiments each; ***, *p* < 0.001, *t* test.

To test this hypothesis, we determined whether sphingosine binds to cardiolipin *in vitro*. To this end, we co-incubated cardiolipin that was immobilized to agarose beads with soluble sphingosine and determined the binding of sphingosine. The results of these co-precipitation experiments show that sphingosine binds cardiolipin (Fig. 5*A*) most efficiently at low pH. Sphingosine showed some residual binding to control lipids, *i.e.* phosphatidylethanolamine or phosphatidylcholine, which was removed by addition of 0.1% NP-40 to reduce hydrophobic interactions between the acyl chains (Fig. 5*A*).

Next, we incubated sphingosine immobilized to beads with soluble cardiolipin. The results confirm the interaction of sphingosine with cardiolipin (Fig. 5*B*).

The binding of sphingosine to cardiolipin depends on the pH and is maximal at pH 6.0 and greatly reduced at pH 8.0. This supports the notion that protonation of the NH_2_ group in sphingosine is required for binding to cardiolipin. The binding assays show an almost quantitative binding of the two lipids at low or physiological pH indicating a strong binding affinity (Fig. 5, *A* and *B*).

To gain insight into potential mechanisms of how sphingosine kills bacteria upon binding to cardiolipin, we generated cardiolipin-containing liposomes and tested binding of sphingosine. These studies demonstrate that cardiolipin-containing liposomes bind sphingosine. Binding of sphingosine to cardiolipin results in a marked decrease of membrane fluidity (Fig. 5*C*). This is consistent with the hypothesis that sphingosine induces rigid, gel-like cardiolipin-enriched domains in bacteria *in vivo*.

**Figure 6.**
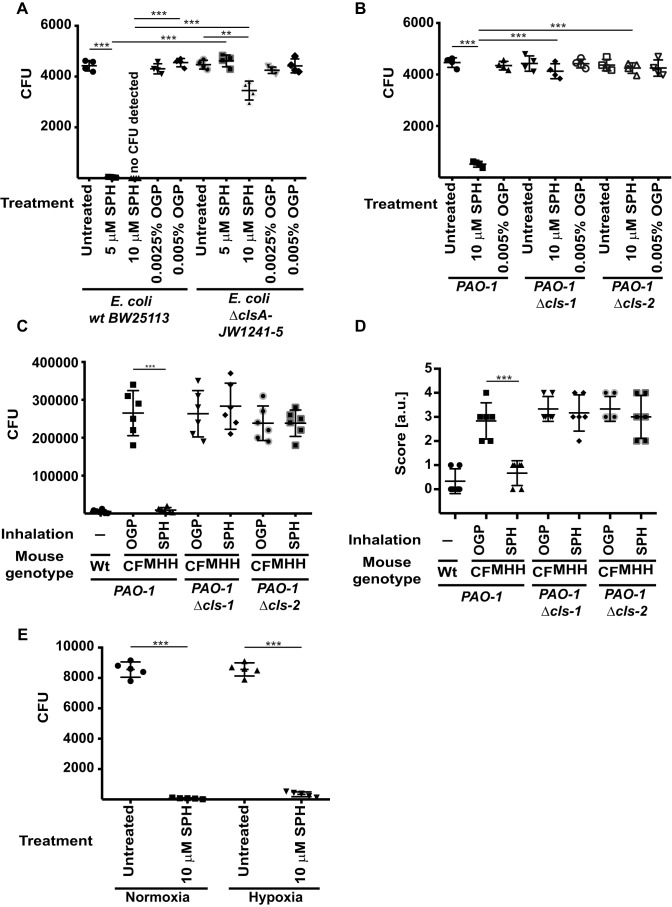
**Sphingosine-mediated killing of bacteria requires cardiolipin.** *A*, each of the 5000 cfu of *E. coli* strain JW1241-5 (with a deletion of cardiolipin synthase A (Δ*clsA*) or the parental WT strain BW25113) was incubated with 5 or 10 μm SPH or with the corresponding concentration of the solvent OGP or left untreated in PBS (pH 7.0) for 60 min. The bacteria were then washed; aliquots were plated on TSB plates, and cfu were counted after growth overnight. Shown are the means ± S.D. of four independent experiments each; **, *p* < 0.01; ***, *p* < 0.001, ANOVA. *B*, WT or cardiolipin synthase-deficient *P. aeruginosa* PAO-1 was treated with 10 or 20 μm sphingosine for 1 h, plated, and grown overnight, and cfu were counted. Displayed are the means ± S.D. of four independent experiments each; *** *p* < 0.001; ANOVA. *C* and *D*, WT mice or CF mice (CF^MHH^) were intranasally infected with 5 × 10^7^ cfu of PAO-1 or the cardiolipin synthase-deficient mutants of PAO-1 and inhaled with 800 μl of a 125 μm SPH suspension in 0.9% NaCl 1 h after the infection, and the numbers of bacteria in the lung (*C*) and the sickness score (*D*) were determined 5 h after the infection. Inhalation with the solvent, *i.e.* 0.0625% OGP in 0.9% NaCl, served as controls. Shown are the means ± S.D. of four independent experiments each; ***, *p* < 0.001; *t* test. *E*, *P. aeruginosa* 762 (10^5^ cfu) was grown and treated with 10 μm sphingosine for 1 h under hypoxic or normoxic conditions in a BD Gas Pak chamber (BD Biosciences), and bacteria were plated, and cfu were determined after overnight growth. Displayed are the means ± S.D. of four independent experiments each; ***, *p* < 0.001, ANOVA.

To demonstrate the role of cardiolipin in sphingosine-mediated killing of bacteria, we used an *E. coli* strain that lacks cardiolipin synthase A. Although *E. coli* expresses three cardiolipin synthases, only cardiolipin synthase A is expressed under logarithmic growth conditions (20). The results show that the *E. coli* strain lacking cardiolipin synthase A is almost completely resistant to sphingosine, whereas the WT strain is rapidly killed (Fig. 6*A*).

These studies were recapitulated with two transposon mutants of *P. aeruginosa* strain PAO-1 that also lack cardiolipin synthase. These strains were resistant to sphingosine-mediated killing *in vitro* (Fig. 6*B*). We also infected mice deficient for Cftr (22) with the WT and the two mutant *P. aeruginosa* strains and determined the effect of an inhalation of sphingosine on pulmonary *P. aeruginosa* counts. These studies reveal that the cardiolipin synthase-deficient *P. aeruginosa* strains are resistant to sphingosine inhalation, whereas the parental strain was killed by inhalation of sphingosine (Fig. 6, *C* and *D*).

In addition, we wanted to exclude the possibility that sphingosine kills bacteria by interfering with the respiratory process only. Therefore, we tested sphingosine efficiency under hypoxic conditions. Hypoxia reduced but did not abrogate the bactericidal effects of sphingosine on *P. aeruginosa* (Fig. 6*E*).

## Discussion

Our studies demonstrate that sphingosine induces a very rapid death of *P. aeruginosa* and *S. aureus* that is obviously caused by a massive increase of membrane permeability, loss of important metabolites such as ATP, and a loss of metabolic activity. These findings are consistent with a previous study that showed rapid membrane permeabilization of *S. aureus* upon incubation with certain fatty acids (23). This study also tested the effects of sphingosine, which is not a fatty acid but rather a sphingoid base, on bacterial killing and reported a minimal inhibitory concentration of 7.8 μm for sphingosine (23), which is very similar to the observations in this study. However, the molecular mechanism of sphingosine-mediated killing of bacteria was not addressed in this study. Additional EM studies demonstrated that incubation of *S. aureus* and *E. coli* with 5 μm sphingosine induces a disruption and even loss of the cell wall of *S. aureus* (15). In contrast, the cytoplasmic membrane and the outer envelope in Gram-negative *E. coli* appeared to be intact after sphingosine incubation. This indicates that sphingosine does not simply lyse bacteria, in accordance with the conclusions of this study.

Here, we demonstrate that the NH_2_ group in sphingosine is critical for mediating bacterial killing and that sphingosine is inactive at alkaline pH. This strongly suggests that the NH_2_ group of sphingosines must be protonated to mediate killing of pathogens. We have previously shown that sphingosine is mainly present on the surface of epithelial cells in the nose, trachea, and bronchi (12, 13, 18), and in fact, the pH on these epithelial cell layers is slightly acidic (19). Thus, sphingosine will be protonated in its natural environment.

These findings led us to speculate that sphingosine binds to negatively-charged lipids in the plasma membrane of bacteria. Furthermore, we noted that inhalation by mice or mini-pigs with even higher doses of sphingosine (up to 3 μmol total) twice daily over 14 days did not result in any damage or inflammation in the trachea, bronchi, or lungs (24, 25). Thus, we further speculated that sphingosine targets a highly negatively-charged lipid in bacterial membranes that is not present in mammalian plasma membranes. Cardiolipin fulfills these criteria because it is highly negatively-charged and present in the plasma membrane of bacteria but absent from the plasma membrane of mammalian cells, in which it is present exclusively in mitochondrial membranes. Furthermore, cardiolipin is critical for respiration, and interference with cardiolipin might also interfere with respiration, although our data show that sphingosine also kills pathogens under hypoxic conditions, suggesting that an interaction of sphingosine with cardiolipin does not kill bacteria by blocking respiration.

Our studies demonstrate that sphingosine binds to cardiolipin; furthermore, they show that the presence of cardiolipin is required for sphingosine-mediated bacterial killing. How does the interaction of sphingosine with cardiolipin mediate death of bacteria? Cardiolipin contains two phosphatidyl residues connected by a glycerol bridge and four associated fatty acyl chains, which are characterized by a high degree of symmetry and unsaturation. This structure results in a relatively small cross-section of its headgroup relative to the large cross-section of its four large tailgroups. Therefore, the incorporation of cardiolipin into membranes results in a large negative curvature (26). Binding of positively-charged sphingosine may trigger an aggregation of negatively-charged cardiolipin molecules. The aggregation of cardiolipin may result in the formation of very rigid, gel-like membrane domains in the otherwise more fluid membrane. The disturbance of the structure of the membrane may ultimately result in membrane permeabilization and thereby in rapid bacterial death. These notions are supported by our studies on liposomes that indicate a marked decrease of membrane fluidity upon binding of sphingosine.

Previous studies have shown that the aminoglycoside antibiotic 3′,6-dinonyl neamine interacts with cardiolipin in bacterial membranes and mediates a clustering of cardiolipin in the bacterial membrane (27). This resulted in decreased fluidity and increased permeability. These data strongly support the notion that binding of positively-charged amphiphilic amines to bacterial membranes results in killing of bacteria by changing the biophysical properties of the bacterial plasma membrane by clustering cardiolipin (27).

Consistent with the notion that positively-charged sphingosine binds to negatively-charged membrane lipids, we noted that higher doses (>20 μm) of sphingosine also killed the *E. coli* strain that was deficient in cardiolipin synthase-A (data not shown). This finding suggests that cardiolipin is the primary target of sphingosine and that, at lower concentrations of sphingosine, the clustering of cardiolipin is sufficient and required to alter bacterial membranes and to kill bacteria. However, sphingosine is also able to cluster smaller negatively-charged lipids, but higher concentrations of sphingosine are necessary for producing large rigid domains that disrupt the membrane structure in the absence of cardiolipin.

The binding of sphingosine to cardiolipin in bacterial membranes also provides an explanation for the finding that inhalation of sphingosine even up to concentrations of 1 mm does not induce any toxicity in epithelial cells. Because mammalian cells contain cardiolipin exclusively in mitochondria, much higher concentrations and internalization of exogenously added sphingosine are required to reach cardiolipin within mitochondria and to affect the viability of mammalian cells. However, because extracellular sphingosine is either integrated into the plasma membrane or endocytosed and thus reaches endosomes, multivesicular bodies, and lysosomes, mitochondria are not easily targeted by extracellularly-applied sphingosine.

In summary, our data demonstrate that sphingosine very rapidly kills *P. aeruginosa* and *S. aureus* at low micromolar concentrations. Mechanistically, we demonstrate that sphingosine binds to cardiolipin in bacterial membranes and exerts thereby its bactericidal effects. These studies provide a mechanism of how sphingosine kills pathogens and justify the further development of sphingosine as a novel antibacterial drug.

## Experimental procedures

### Bacterial strains

We used the *P. aeruginosa* strains 762 and ATCC 27853, the septic *S. aureus* strain DH, and the *E. coli* strains BW25113 JW1241-5. The *P. aeruginosa* strain 762 and the *S. aureus* strain DH are clinical isolates (13, 21), and the *P. aeruginosa* strain ATCC 27853 is a laboratory strain. The *E. coli* strain BW25113 is a WT strain, and the JW1241-5 strain has a deletion of cardiolipin synthase A (Δ*cls*). The *E. coli* strains were obtained from the *E. coli* stock center of Yale University. Two cardiolipin synthase *(*Δ*cls*)-deficient *P. aeruginosa* strains that were generated from PAO-1 by transposon-mediated mutagenesis were obtained from the transposon mutant collection at the University of Washington, Seattle. All *P. aeruginosa* strains and *E. coli* strains BW25113 and JW1241-5 were grown overnight on tryptic soy agar (TSA; BD Biosciences, Heidelberg, Germany). *S. aureus* DH was grown overnight on blood agar plates. The bacteria were then transferred to tryptic soy broth (TSB, BD Biosciences); the density was adjusted to an OD_550 nm_ of 0.2–0.25, and the bacteria were grown for 1 h at 37 °C with 125 rpm shaking to reach the early logarithmic phase and provide reproducible growth conditions. Bacteria were then centrifuged at 1710 × *g* (2800 rpm) (*P. aeruginosa* and *E. coli*) or 2240 × *g* (3000 rpm) (*S. aureus*) for 10 min, washed once in sterile HEPES/saline (H/S: 132 mm NaCl, 20 mm HEPES (pH 7.4), 5 mm KCl, 1 mm CaCl_2_, 0.7 mm MgCl_2_, 0.8 mm MgSO_4_), and resuspended in H/S for the following assays.

### Sphingosine

Sphingosine (Avanti Polar Lipids, Alabaster, AL) was resuspended as a 20 mm stock solution in distilled water containing 10% octylglucopyranoside (Sigma, Deisenhofen, Germany). Prior to use, the sphingosine stock was sonicated in a bath sonicator for 10 min to promote the formation of micelles.

### Cell permeability

Each 10^5^ cfu of *P. aeruginosa* strains 762 and ATCC 27853 or *S. aureus* strain DH were incubated in H/S for 5 min with sphingosine at a concentration of 1 or 10 μm. Octylglucopyranoside, the solvent of sphingosine, was added at the same concentrations as in the samples with sphingosine. If indicated, either 0.1% Triton or 80 μg/ml nisin (Sigma, Deisenhofen, Germany) was added as positive controls for membrane permeabilization. Bacteria were then incubated with 100 nm TO-PRO-3 iodide (Life Technologies, Inc.) at room temperature for 5 min. TO-PRO-3 is a monomeric carbocyanine, staining dsDNA and thereby detecting permeabilization of the bacteria. Bacteria were pelleted and analyzed by flow cytometry using a FACSCalibur (BD Biosciences) counting 20,000 bacteria/sample.

### ATP measurements

Each of the 10^5^ cfu *P. aeruginosa* strains 762 and ATCC 27853 or the *S. aureus* strain DH was incubated in H/S for 60 min with sphingosine at a concentration of 1 or 10 μm. Octylglucopyranoside was added at the same concentrations as in the samples with sphingosine. Bacteria were pelleted by centrifugation for 10 min at 2240 × *g*, and the release of ATP from the bacteria into the supernatant/medium and the remaining ATP in the resuspended pellet was measured with the BacTiter-Glo reagent (Promega) according to the instructions of the manufacturer. The pellet was resuspended in PBS and treated with 0.1% Triton to permeabilize bacteria. The samples were incubated for 2 min and 250 rpm horizontal shaking, and the luminescence was measured using a luminescence reader. Either 0.1% Triton or 80 μg/ml nisin was added as a positive control for membrane permeabilization and ATP release into the medium. ATP concentration was determined using a standard curve of ATP. Protein concentrations were determined in aliquots of the incubations prior to centrifugation using a commercial Bradford assay (Bio-Rad).

### Metabolic activity of P. aeruginosa

Each of the 10^5^ cfu *P. aeruginosa* strains 762 and ATCC 27853 or *S. aureus* strain DH was incubated in H/S for 15 min with sphingosine at a concentration of 1 or 10 μm. Octylglucopyranoside was added at the same concentration as in the samples with sphingosine. Metabolic activity was determined using the Vybrant Cell Metabolic Assay Kit (Life Technologies Inc., Darmstadt, Germany). To this end, bacteria were incubated with 1 μm C_12_-resazurin for an additional 15 min, washed, and analyzed by flow cytometry. The assay employs the conversion (reduction) of the nonfluorescent C_12_-resazurin to fluorescent C_12_-resorufin by viable cells. Either 0.1% Triton or 80 μg/ml nisin was added as a positive control for membrane permeabilization.

### Interaction of cardiolipin, phosphatidylcholine, and phosphatidylethanolamine with sphingosine and measurements of sphingosine by sphingosine kinase assay

Agarose-bound cardiolipin, phosphatidylcholine, phosphatidylethanolamine, or control beads were purchased from Echelon Biosciences (Salt Lake City, UT). Aliquots of 30 μl were co-incubated with 1 nmol of either sphingosine or octylglucopyranoside in 100 μl of H/S for 60 min at room temperature. The beads were extensively washed six times in H/S and extracted in CHCl_3_, CH_3_OH, 1 n HCl (100:200:1, v/v/v). Samples were dried and then resuspended in 50 mm HEPES (pH 7.4), 250 mm NaCl, 30 mm MgCl_2_, 1 mm ATP, 10 μCi of [γ-^32^P]ATP and 0.01 unit/ml sphingosine kinase 1 (R&D Systems). Samples were incubated for 30 min at 37 °C with shaking (350 rpm). The sphingosine kinase reaction was terminated by adding 100 μl of H_2_O, followed by the addition of 20 μl of 1 n HCl, then 800 μl of CHCl_3_, CH_3_OH, 1 n HCl (100:200:1, v/v/v), and 240 μl each of CHCl_3_ and 2 m KCl. The lower phase was collected, dried, dissolved in 20 μl of CHCl_3_, CH_3_OH (1:1, v/v), and separated on Silica G-60 thin-layer chromatography (TLC) plates (Merck) with CHCl_3_, CH_3_OH, acetic acid, H_2_O (90:90:15:5, v/v/v/v) as developing solvent. The TLC plates were analyzed with a phosphorimager, and sphingosine was quantified with a standard curve. The solvent octylglucopyranoside (used as a control) showed no background binding to cardiolipin in the kinase assays.

### Interaction of sphingosine with cardiolipin

Agarose-bound sphingosine or control beads were purchased from Echelon Biosciences. Aliquots of 20 μl were co-incubated with 2 nmol of either cardiolipin or, as control, DMSO in 200 μl H/S at different pH values (pH 5.0, 6.0, 7.0, 8.0, and 9.0) for 30 min at room temperature. The beads were extensively washed six times in H/S and extracted in CHCl_3_,CH_3_OH, 1 n HCl (100:100:1, v/v/v). Samples were dried and then resuspended in 10 μm cardiolipin probe (BioVision, Biozol Diagnostica, Eching, Germany), a fluorescent probe for the detection and quantification of cardiolipin. The samples were incubated for 5 min and 250 rpm shaking, and the fluorescence was measured using a fluorescence reader. Cardiolipin concentration was determined using a standard curve of cardiolipin.

### Bacterial killing assays

Each of the 10,000 cfu *P. aeruginosa* strains 762, ATCC 27853, PAO-1, or of the two cardiolipin synthase (Δcls)-deficient PAO-1 mutants or the *S. aureus* strain DH or 5000 cfu *E. coli* strains BW25113 or JW1241-5 that were grown to the early logarithmic phase, as described above, was incubated with 1, 5, or 10 μm stearylamine or sphingosine or with the corresponding concentrations of the solvent octylglucopyranoside in H/S or phosphate-buffered saline (PBS) for the indicated times. If indicated, the pH of the PBS was varied from pH 6.0 to 7.0 and 8.0. The bacteria were then washed; aliquots were plated on TSB plates, and cfu were counted after growth overnight.

### Liposome studies

Membrane fluidity of different liposomes before and after addition of 10 μm sphingosine was evaluated by measuring the fluorescence intensity of 1,6-diphenyl-1,3,5-hexatriene (DPH; Sigma-Aldrich) incorporated in the liposome membrane. Liposomes were prepared by high-pressure extrusion immediately before use. Briefly, to achieve the desired composition of the different liposomes (see Fig. 5*C*; lipid composition is indicated in the figure in mol %), stock solutions of the indicated lipids (l-α-phosphatidylcholine, Avanti Polar Lipids; cholesterol, Echelon Biosciences; sphingomyelin, Avanti Polar Lipids; and cardiolipin, Avanti Polar Lipids) (dissolved in chloroform; 10 mm) were mixed accordingly and dried by rotary evaporation under vacuum. After the solvent removal, dried lipid mixtures were hydrated in 10 mm HEPES, 100 mm NaCl buffer (pH 7.4) at 50 °C with periodic agitation on a thermal mixer. The liposome solution was frozen at −80 °C and thawed at 50 °C. This freeze-thaw cycle was performed five times. The resulting multilamellar vesicles (MLVs) were used to prepare large unilamellar vesicles (LUVs) by passing the MLV suspension 11 times through 100-nm polycarbonate filters in an extruder device (Avanti Mini-Extruder; Avanti Polar Lipids, Alabaster, AL). Indicated LUVs were incubated with 10 μm sphingosine for 1 h at 40 °C with periodic agitation on a thermal mixer. A 1 mm stock solution of the fluorescent probe DPH was prepared in dimethyl sulfoxide (DMSO; Sigma-Aldrich), and a working solution of 10 μm (in PBS) was added to the LUV suspension in a ratio of 200:1 lipid/DPH. Fluorescence intensity of DPH was measured using a fluorescence reader (FLUOstar Omega; BMG Labtech) after incubation at 40 °C for 1 h and 250 rpm shaking in the dark.

### Isolation and treatment of mitochondria

To isolate mitochondria, cells were incubated for 30 min at 4 °C in 0.3 m sucrose, 10 mm TES (pH 7.4), and 0.5 mm EGTA. Cells were then Dounce-homogenized, and nuclei and unbroken cells were pelleted by centrifugation for 5 min at 600 × *g* at 4 °C. Supernatants were collected and centrifuged at 6000 × *g* for 10 min at 4 °C. The pellets were resuspended, and mitochondria were purified employing a 10-min 60, 30, and 18% Percoll gradient centrifugation at 8500 × *g* at 4 °C. Mitochondria at the interface between the 30 and 60% layers were collected, washed twice, and resuspended in 50 mm PIPES-KOH (pH 7.0 or 8.0), 50 mm KCl, 2 mm MgCl_2_, 2 mm EGTA, 10 μg/ml aprotinin/leupeptin, 2 mm ATP, 10 mm phosphocreatine, 5 mm succinate, and 50 μg/ml creatine kinase (buffer 1). Mitochondria (corresponding to 1 × 10^6^ cells) were incubated on ice for 30 min with 0.5 μm sphingosine at pH 7.0 or 8.0 or left untreated. The mitochondria were then centrifuged; supernatants were discarded; the mitochondria were resuspended at 37 °C in prewarmed buffer 1 and incubated for 10 min at 37 °C to permit release of cytochrome *c*. The reaction was terminated by addition of 1 volume of ice-cold buffer 1, centrifuged at 20,800 × *g* at 4 °C, and then supernatant was added to 5× SDS-sample buffer. The samples were analyzed for cytochrome *c* release by Western blotting. Western blottings were analyzed using a monoclonal mouse anti-cytochrome *c* antibody (clone 7H8.2C12, BD Pharmingen) and an ECL system.

### Hypoxia experiments

*P. aeruginosa* 762 was grown under hypoxic or normoxic conditions in a BD Gas Pak chamber (BD Biosciences) for 24 h. 10^5^ cfu were then exposed to 10 μm sphingosine for 1 h. Hypoxia or normoxia was maintained during the incubation period. cfu were then determined to measure the survival of the bacteria.

### Mouse infections

B6.129P2(CF/3)-*Cftr^TgH(neoim)Hgu^* (abbreviated *Cft*r*^MHH^*) mice were used in this study (22). These mice express low levels of Cftr allowing feeding of a normal diet as described previously. Mice were housed in the Central Laboratory Animal Facility of the University of Duisburg-Essen, Germany. We used female and male mice at an age of at least 16 weeks and a weight between 25 and 35 g. Mice were divided into cages of equal size (usually 3–4 mice) by animal unit technical staff with no involvement in study design. Cages were randomly assigned to an experimental group. The investigators were blinded to the group allocation during the experiment and/or when assessing the outcome.

PAO-1 or one of the two Δ*cls*-PAO-1 mutants were grown to the early logarithmic phase as described above and resuspended in RPMI 1640 medium plus 10 mm HEPES to a final concentration of 5 × 10^6^ cfu in 20 μl of medium. To infect mice with the *P. aeruginosa* strains, mice were lightly anesthetized with diethyl ether, which does not affect ciliary function (22, 28). The mice were then inoculated intranasally with 5 × 10^7^ cfu of each *P. aeruginosa* strain employing a plastic-coated 30-gauge needle, which was inserted 2 mm into the nose. Mice then inhaled 800 μl of a 125 μm sphingosine suspension in 0.9% NaCl 1 h after the infection (the mice inhaled ∼10% of the inhalation volume). We determined the health status of the mice 5 h after infection using the following score: sickness score 0, unaffected (healthy appearance); score 1, slightly affected (ruffled fur); score 2, moderately affected (ruffled fur, breathing slightly impaired, normal body temperature); score 3, severely affected (ruffled fur, heavy breathing, lower body temperature). Bacterial numbers were then determined in the mouse lungs 5 h after infection. To this end, mice were sacrificed, and the lungs were removed, homogenized, and lysed in 5 mg/ml saponin to release intracellular bacteria. Bacteria were then pelleted at 2240 × *g*, washed once in sterile PBS, diluted, plated, and grown in duplicate on TSA plates for 12 h. Bacterial numbers were counted. Infection experiments were approved by the Bezirksregierung Duesseldorf, Duesseldorf, Germany, under permission number 81-02.04.2019.A134. The actual care and treatment of the animals were performed and/or overseen by veterinarians of the Central Animal Facility of the University Hospital Essen, Essen, Germany.

### Statistics

Data are expressed as arithmetic means ± S.D. To compare more than two groups, we used one-way ANOVA followed by post hoc Student's *t* tests for all pairwise comparisons applying Bonferroni correction for multiple testing. The *p* values for the pairwise comparisons were calculated after Bonferroni correction. If only two groups were compared, we used the Student's *t* test. All values were tested for normal distribution. The sample size planning for the continuous variables in *in vivo* infection experiments was based on two-sided Wilcoxon–Mann-Whitney tests (software: G*Power, Version 3.1.7, from the University of Duesseldorf, Germany). Investigators were blinded for histology experiments and animal identity.

## Data availability

All data are given in the paper.
